# Controlling Obesity and Metabolic Diseases by Hydrodynamic Delivery of a Fusion Gene of Exendin-4 and α1 Antitrypsin

**DOI:** 10.1038/s41598-019-49757-y

**Published:** 2019-09-17

**Authors:** Mingming Gao, Dexi Liu

**Affiliations:** Department of Pharmaceutical and Biomedical Sciences, College of Pharmacy, University of Georgia, 250 West Green Street, Athens, GA 30602 USA

**Keywords:** Obesity, Molecular medicine

## Abstract

Obesity and associated metabolic comorbidities represent a growing public health problem. In this study, we demonstrate the use of a newly created fusion gene of exendin-4 and α1-antitrypsin to control obesity and obesity-associated metabolic disorders including insulin resistance, fatty liver and hyperglycemia. The fusion gene encodes a protein with **e**xendin-4 peptide placed at the N-terminus of human α-1 **a**nti**t**rypsin, and is named *EAT*. Hydrodynamic transfer of the *EAT* gene to mice prevents high-fat diet-induced obesity, insulin resistance and fatty liver development. In diet-induced obese mice, expression of *EAT* gene induces weight loss, improves glucose homeostasis, and attenuates hepatic steatosis. In *ob/ob* mice, *EAT* gene transfer suppresses body weight gain, maintains metabolic homeostasis, and completely blocks fatty liver development. Six-month overexpression of the *EAT* fusion gene in healthy mice does not lead to any detectable toxicity. Mechanistic study reveals that the resulting metabolic benefits are achieved by a reduced food take and down-regulation of transcription of pivotal genes responsible for lipogenesis and lipid droplet formation in the liver and chronic inflammation in visceral fat. These results validate the feasibility of gene therapy in preventing and restoring metabolic homeostasis under diverse pathologic conditions, and provide evidence in support of a new strategy to control obesity and related metabolic diseases.

## Introduction

Obesity (body mass index > 30) has become a major public health issue in recent years. The prevalence of obesity in the US is ~35.5% and ~35.8% among adult men and women, respectively^1^. This disease is linked to a number of severe metabolic comorbidities such as diabetes, nonalcoholic fatty liver disease (NAFLD), and cardiovascular events^1,2^. Behavior interventions can lead to metabolic benefits in the general population, but increasing evidence demonstrates that these interventions are essential but not sufficient to maintain a healthy weight, particularly in individuals predisposed to obesity^3–5^. It remains difficult to establish a new strategy for effective management of these aforementioned metabolic diseases.

This difficulty stems primarily from the complex pathophysiology of obesity which includes excess food intake and chronic inflammation^6–8^. Food intake is controlled by the gut-brain axis, in which glucagon-like peptide-1 (GLP-1) plays a dominant role^9^. GLP-1 is made by intestine L-cells in response to food ingestion and released into circulation^9^, which subsequently increases pancreatic insulin secretion and inhibits gastric emptying in the stomach. Reduction in GLP-1 release has been shown in obese patients, resulting in excess energy intake and imbalance of metabolic homeostasis^9–11^. In addition to excess energy intake, another typical feature of morbid obesity is chronic inflammation which plays an indispensable role in the initiation and progression of obesity-related metabolic complications^7,12,13^. In fact, without tissue inflammation, weight gain alone would not lead to severe metabolic complications such as glucose intolerance and hepatic fat aggregation^14–16^. Simultaneous repressing energy intake and relieving inflammation is expected to reduce body weight, promote adipose remodeling, and lead to restoration of metabolic homeostasis.

In this study, we created a novel fusion gene for pharmacological intervention of obesity and related metabolic disorders. The fusion gene contains the sequence of exendin-4 (Ex4), a potent agonist of the GLP-1 receptor, placed at the 5′ end of human α-1 antitrypsin (hAAT) gene. Anti-inflammation activity of AAT has been well-recognized and previously shown^17–19^. We abbreviated the fusion gene as *EAT* by taking the critical letters from *Ex4* and *AAT*. We assessed the preventive and therapeutic effects of EAT on obesity, glucose homeostasis and hepatic steatosis in both dietary obese mice and genetically modified *ob/ob* mice. In this report, we provide evidence in support of the feasibility of a gene therapy-based strategy to manage obesity and obesity-associated metabolic disorders.

## Results

### Design and construct of plasmid vectors for *EAT* expression

**Figure 1:**
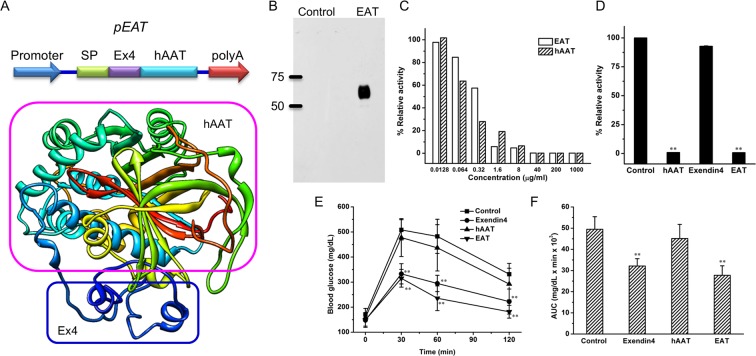
Schematic presentation of plasmid constructs and validation of elastase inhibitor activity and exendin-4 activity of recombinant EAT. (**A**) Schematic presentation of pEAT construct and predicted structure of EAT protein based on PHYRE2 computer software. SP = signal peptide, Ex4 = exendin-4, hAAT = human α-1 antitrypsin. (**B**) Western blotting of purified EAT protein (full-length blot is presented in Supplementary Fig. 1). (**C**) Inhibition of elastase enzyme activity by hAAT and recombinant EAT protein. Purified proteins were diluted at different concentrations and added to the reaction mixture. The excitation and emission wavelength was 400 and 505 nm respectively. (**D**) Comparison of elastase inhibition activity of different components in EAT. Proteins and peptides were diluted using assay buffer to a final concentration of 20 nmol/ml. A fluorescence-based enzymatic assay was performed following the protocol provided with the kit. Data represent the average of 3 independent experiments. (**E**) Effect of components in EAT on glucose clearance in glucose tolerance test. HFD-induced obese mice (~50 g, n = 5 each group) were pretreated with a single intraperitoneal injection of saline, exendin-4, hAAT or EAT at 20 nmol/kg. A standard IPGTT was carried out 30 min after protein injection. Blood glucose levels were measured at 0, 30, 60 and 120 min after glucose injection. (**F**) Area under the curves of glucose tolerance test in (**E**). Values in (**C–F**) represent average ± SD. **P < 0.01 compared with the control.

Figure 1A shows the design of plasmid vectors employed in the study, including plasmids containing promoter, signal peptide for protein secretion, sequence of *Ex4*, a linker, *hAAT* coding sequence, and polyA signal. A His6 tag was added to the C-terminus of the fusion protein. A computer-based program^20^ predicts that the EAT fusion protein has a globular structure with the secondary structure of each unit conserved. To verify whether the functions of hAAT and Ex4 are preserved in fusion protein, pEAT plasmids were transfected into HEK293T cells using branched polyethylenimine (PEI) as a transfection reagent and EAT recombinant proteins were purified using Ni-NTA affinity chromatography. Figure 1B shows that EAT protein was efficiently purified and confirmed by Western Blotting using an anti-hAAT antibody. Florescence-based proteinase assay shows that purified EAT protein has comparable activity to that of pure hAAT protein in inhibiting elastase activity (Fig. 1C), while native Ex4 peptide showed no activity at equal molar level (Fig. 1D). Glucose tolerance test was employed to assess the activity of Ex4 in EAT. Comparing to control animals and those pre-injected with hAAT, animals pre-injected with either Ex4 peptide or EAT protein showed a much lower level of blood glucose (Fig. 1E) in glucose tolerance test. AUC analysis (Fig. 1F) showed both EAT and Ex4 protein induced ~40% decrease in blood glucose level, suggesting a full preservation of Ex4 activity in EAT.

### *EAT* gene transfer blocks high-fat diet-induced weight gain, hyperadiposity, insulin resistance, fatty liver development, and the expression of relevant genes

**Figure 2:**
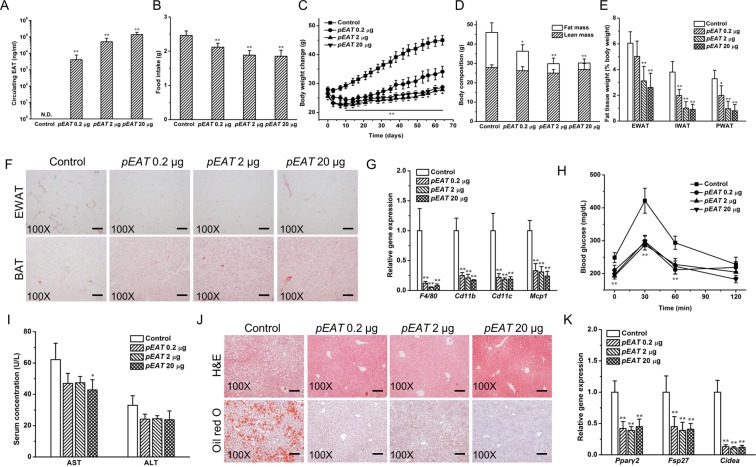
*EAT* gene transfer blocks HFD-induced weight gain, hyperadiposity, macrophage activation, fatty liver development and improves glucose homeostasis. (**A**) Blood levels of EAT protein at 9 weeks after pEAT injection. (**B**) *EAT* gene transfer reduced daily HFD intake of each mouse. (**C**) *EAT* gene transfer repressed HFD-induced weight gain. (**D**) *EAT* gene transfer reduced fat mass while showing no significant impact on lean mass. (**E**) *EAT* gene transfer reduced the weights of white fat depots. EWAT, epididymal white adipose tissue; IWAT, inguinal white adipose tissue; PWAT, perirenal white adipose tissue. (**F**) Representative images of H&E staining of EWAT and BAT; Scale bar = 50 μm. (**G**) Relative mRNA levels of key genes responsible for chronic inflammation in EWAT. (**H**) Profiles of blood glucose concentration as function of time upon intraperitoneal injection of glucose. (I) Blood levels of AST and ALT. (**J**) Representative images of H&E staining and Oil red O staining of the liver sections; Scale bar = 50 μm. (**K**) Relative mRNA levels of key genes responsible for lipogenesis in the liver. Values in (**A–E)**, **(G–I)**, and **(K**) represent average ± SD (n = 5). *P < 0.05 compared with the control, **P < 0.01 compared with the control.

The impacts of *EAT* gene transfer on high fat diet (HFD)-induced weight gain, glucose homeostasis, and fatty liver development were examined with 4 groups of animals. Animals were hydrodynamically injected with 20 µg/mouse of pLIVE empty plasmid (control), or pEAT plasmid at 0.2 µg, 2.0 µg or 20.0 µg per mouse and fed an HFD for 9 weeks. Results in Fig. 2A show that, at the end of the experiment, the blood concentration of EAT protein is ~138 µg/ml in animals injected with 20.0 µg pEAT plasmid DNA per mouse, compared to ~50 µg/ml and ~4 µg/ml with injection of 2.0 or 0.2 µg, respectively. The average food intake of animals (Fig. 2B) decreased with increasing the amount of pEAT plasmid. Control animals consumed 2.46 ± 0.13 g of HFD per day per animal compared to 2.12 ± 0.11 g with 0.2 µg, 1.89 ± 0.13 g with 2.0 µg, and 1.85 ± 0.17 g with 20.0 µg of animals with injection of pEAT plasmid. The average body weight of control mice is 45.0 g compared to 34.1 g at a dose of 0.2 µg of pEAT plasmid DNA (Fig. 2C). There is no weight difference between animals injected with 2.0 and 20.0 µg pEAT plasmid. Magnetic resonance imaging (MRI) analysis (Fig. 2D**)** shows that the average lean mass of all animals is similar but the fat mass is different with control animals exhibiting the highest amount at ~18.2 g, followed by that (~10.1 g) of animals injected with 0.2 µg of pEAT plasmid. Approximately 4.9 g and 3.4 g of fats were seen in animals injected with 2.0 µg or 20.0 µg of pEAT plasmid, respectively. White adipose tissue (WAT) was collected and weighted. Results in Fig. 2E show the highest amount of WAT in control mice, followed by animals injected with 0.2 µg of pEAT plasmid and the animals with the injection dose of 2.0 µg or 20.0 µg per mouse showed the same and lowest WAT. The Hematoxylin and Eosin (H&E) staining of adipose tissue sections revealed the presence of crown-like structures (CLSs) in the white adipose tissue of control animals (Fig. 2F), not in animals with *EAT* gene transfer. More fat content was also seen in brown adipose tissue (BAT) of control animals comparing to that of pEAT injected animals. The presence of macrophages in WAT as indicated by CLS is confirmed by quantitative polymerase chain reaction (qPCR) analysis of macrophage marker genes. Figure 2G shows lower mRNA levels of *F40/80*, *Cd11b*, *Cd11c* and *Mcp1* in pEAT-treated animals comparing to those of the control. Results of glucose tolerance tests show improved glucose homeostasis in pEAT-treated mice (Fig. 2H). The impact of *EAT* gene transfer on liver damage was assessed by the serum concentrations of aspartate aminotransferase (AST) and alanine aminotransferase (ALT). Concentrations of liver specific enzymes (Fig. 2I) were higher in control mice (62 ± 10 U/L, AST; 33 ± 6 U/L, ALT) comparing to those of animals injected with pEAT plasmid DNA (42 ± 7 U/L, AST; 24 ± 5 U/L, ALT). HFD-induced hepatic fat accumulation was also blocked by *EAT* gene transfer as shown in Fig. 2J by both H&E and Oil-red O staining. Vacuole type structures seen in liver sections of control mice are not evidenced in mice received *EAT* gene transfer. No obvious fat accumulation was detected by Oil-red O staining in pEAT-treated animals compared to the control. Results from qPCR analysis of total RNAs extracted from the livers (Fig. 2K) show a decrease in mRNA level of genes responsible for lipogenesis including *Pparγ2*, *Fsp27*, and *Cideα* in animals injected with pEAT plasmid. These results demonstrate that a dose of 2.0 µg or greater of pEAT plasmid is sufficient to block HFD-induced weight gain and obesity-associated macrophage activation, glucose resistance, and fatty liver development.

### *EAT* gene transfer reduces adiposity and improves fatty liver in high-fat diet-induced obese mice

**Figure 3:**
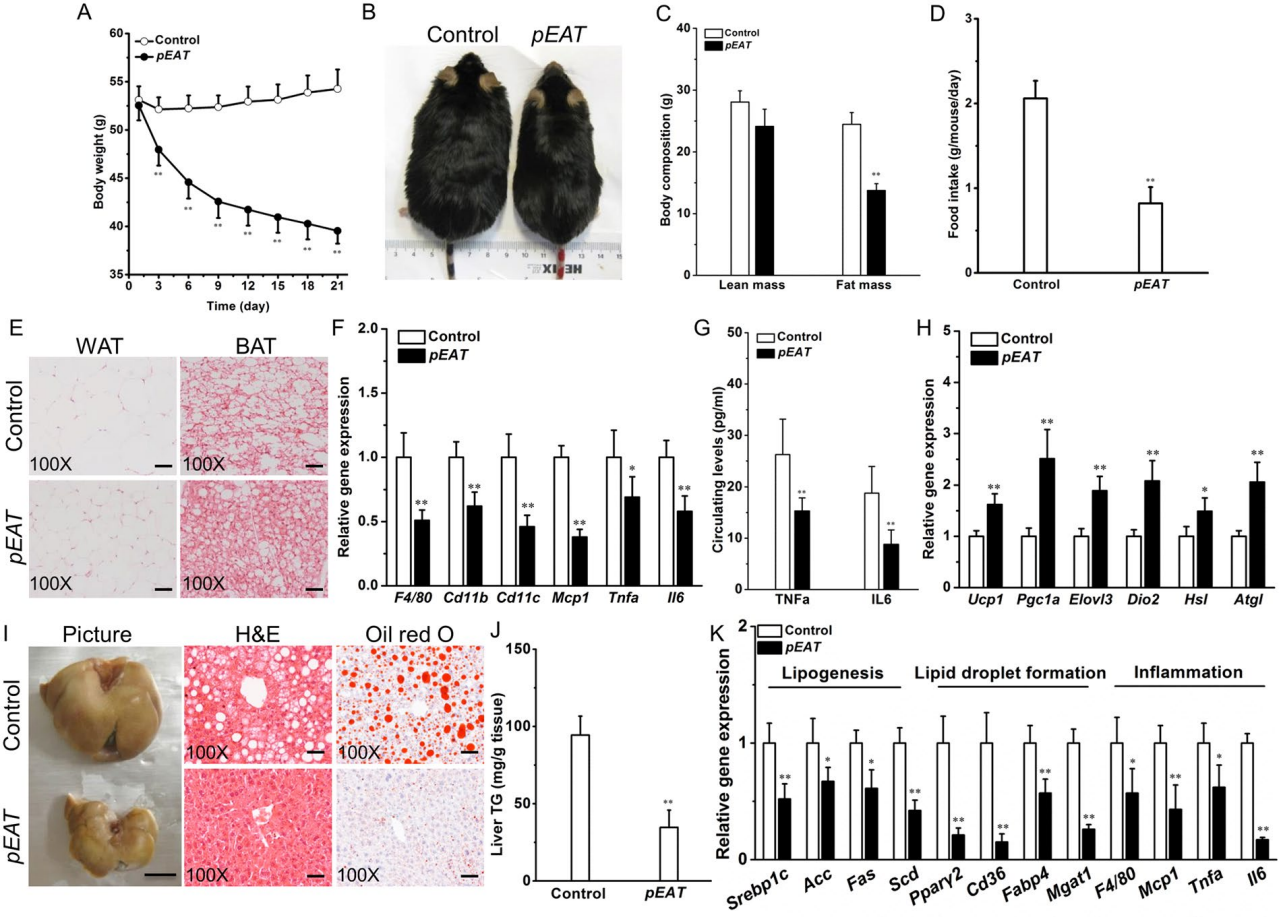
*EAT* gene transfer reduces body weight and improves fatty liver in C57BL/6 mice with diet-induced obesity. Obese mice were kept on high-fat diet and hydrodynamically injected with 20 µg of either pEAT or pLIVE empty plasmid (control). Animal body weight was monitored continuously for 21 days, at which time animals were sacrificed for tissue collection and histological and biochemical analysis. (**A**) Effect of gene transfer on body weight. (**B**) Representative images of mice at the end of experiment. (**C**) Comparative body composition of animals with pEAT or control plasmid transfer. (**D**) Average daily food intake. (**E**) Representative images of H&E staining of WAT and BAT sections; Scale bar = 50 μm. (**F**) mRNA levels of key genes responsible for chronic inflammation in WAT. (**G**) Circulating levels of TNFα and IL6. (H) Expression levels of genes controlling adaptive thermogenesis in brown fat. (**I**) Representative images of the liver and liver tissue sections stained by H&E and Oil red O; Bar = 1 cm for gross image; Scale bar = 50 μm for tissue sections. (**J**) Relative level of triglyceride in the livers. (**K**) Transcription levels of pivotal genes responsible for lipogenesis, lipid droplet formation, and inflammation in the liver. Values in (**A**,**C**,**D**,**F–H**,**J**,**K**) represent average ± SD (n = 5). *P < 0.05 compared with the control; **P < 0.01 compared with the control.

To systematically study the therapeutic effects of *EAT* gene transfer, we hydrodynamically transferred the pEAT plasmids into C57BL/6 mice with HFD-induced obesity, a well-validated and widely used model for obesity and related metabolic studies. Figure 3A shows that transfer of the *EAT* gene progressively reduced body weight in these mice. One single injection of the pEAT plasmid resulted in a weight loss of ~24.7% within 3 weeks (Fig. 3A,B). Body composition determination (Fig. 3C) revealed that the difference in body weight primarily resulted from reduction in fat mass (by ~43.1%) and to a lesser degree from lean mass (~12.8%). *EAT* gene transfer substantially repressed the average food intake by ~60.2% (Fig. 3D). *EAT* gene transfer greatly reduced adipocyte hypertrophy in WAT and decreased fat deposition in BAT (Fig. 3E). *EAT* gene transfer also down-regulated transcription of pivotal genes involved in WAT chronic inflammation, including *Tnfa* and *Il6* (Fig. 3F). In line with the gene expression data, protein levels of TNFα and IL6 in blood were decreased by ~41.7% and ~53.2%, respectively, in pEAT injected mice compared to the control group (Fig. 3G). Thermogenic genes in BAT were up-regulated by *EAT* gene transfer (Fig. 3H). The treatment reversed fatty liver (Fig. 3I) and reduced hepatic fat content by ~63.5% (Fig. 3J). The reduced hepatic fat deposition was associated with a decrease in transcriptional levels of the genes involved in lipid synthesis, lipid droplet formation and inflammation (Fig. 3K).

### *EAT* gene transfer improves glucose homeostasis in obese mice

**Figure 4:**
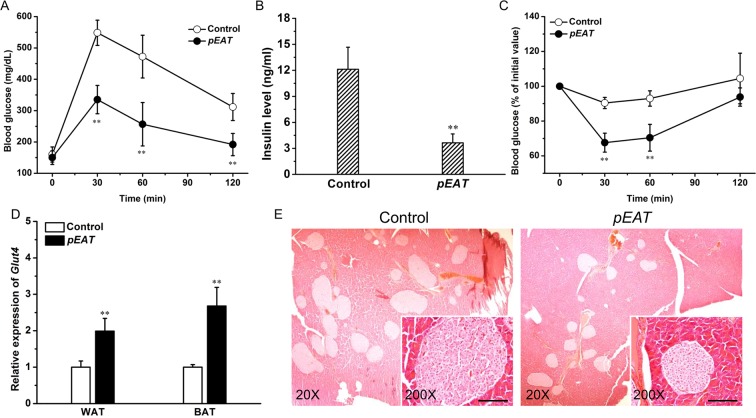
*EAT* gene transfer improves glucose tolerance and alleviates insulin resistance in obese C57BL/6 mice. Obese C57BL/6 mice were hydrodynamically transferred with pEAT or control plasmids. Animals were fasted for 6 h on day 15 and intraperitoneal injection of glucose solution was performed. Animals were fasted for 4 h before intraperitoneal injection of insulin solution on day 18. Blood samples were collected after insulin or glucose injection from mouse tails at different time and blood glucose concentrations were determined. (**A**) Profiles of blood glucose concentration as function of time upon intraperitoneal injection of glucose. (**B**) Serum concentration of insulin at the end of 21-day experiment. (**C**) Profiles of glucose concentration (percentage of initial value) as a function of time upon intraperitoneal injection of insulin. (**D**) mRNA levels of *Glut4* in WAT and BAT. (**E**) Representative images of H&E staining of the pancreatic islets; Scale bar = 50 μm. Values in (**A–D**) represent average ± SD (n = 5). **P < 0.01 compared with the control.

We next assessed the impact of *EAT* gene transfer on glucose metabolism in HFD-induced obese mice. Figure 4A shows that *EAT* gene transfer markedly promoted glucose tolerance in these obese mice. The peak glucose levels for mice with or without *EAT* expression were ~335.2 and ~548.4 mg/dL, respectively, and the area under the curve (AUC) was significantly reduced by ~69.9% in mice with *EAT* gene transfer. *EAT* gene transfer also reduced blood insulin levels (Fig. 4B), indicating improved insulin sensitivity. This notion was further supported by the results of insulin tolerance test, which showed that the mice with *EAT* gene transfer had a much stronger response to insulin injection (Fig. 4C). To study the underlying mechanism, we measured mRNA levels of *Glut4* in adipose tissues. Figure 4D shows that *EAT* gene transfer increased *Glut4* expression in WAT and BAT by 1.9-fold and 2.7-fold, respectively. In alignment with the improved insulin sensitivity, *EAT* gene transfer markedly reduced islet hypertrophy (Fig. 4E).

### *EAT* gene transfer suppresses adiposity and improves glucose metabolism in leptin-deficient *ob/ob* mice

**Figure 5:**
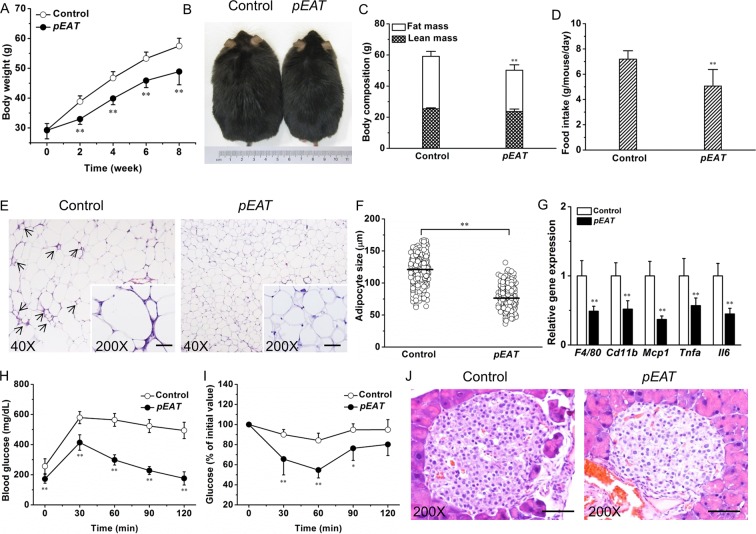
Effects of *EAT* gene transfer on transgenic *ob/ob* mice. (**A**) Body weight change. (**B**) Representative images of mice at the end of the experiment. (**C**) Body composition. (**D**) Food intake. (**E**) Representative images of H&E staining of WAT sections. Arrows point to crown-like structures. (**F**) Adipocyte size distribution. (**G**) Expression levels of key genes responsible for chronic inflammation in WAT. (**H**) Profiles of blood glucose concentration as function of time upon intraperitoneal injection of glucose. (**I**) Profiles of glucose concentration (percentage of initial value) as a function of time upon intraperitoneal injection of insulin. (**J**) Representative images of H&E staining of the pancreatic islets; Scale bar = 50 μm. Values in (**A**,**C**,**D**) and (**F–I**) represent average ± SD (n = 5). *P < 0.05 compared with the control; **P < 0.01 compared with the control.

We next determined the activity of *EAT* gene transfer in the context of leptin deficiency on *ob/ob* mice. Overexpression of the *EAT* gene suppressed weight gain by ~8.5 g (Fig. 5A,B). The suppressed weight gain was mainly contributed by a less increase in fat mass (Fig. 5C), which was correlated with a decrease in food intake (Fig. 5D). Histological examination of visceral fat revealed that *EAT* gene transfer suppressed adipocyte hypertrophy and blocked the development of CLS in WAT (Fig. 5E). Further quantification demonstrated that the average size of adipocytes in mice with *EAT* gene transfer was ~36.5% smaller than that of the control (Fig. 5F). Transcription levels of key inflammatory genes including *F4/80*, *Cd11b*, *Mcp1*, *Tnfa*, and *Il6* were significantly lower in mice with *EAT* gene transfer comparing to the control (Fig. 5G). Consistent with the repressed weight gain and attenuated adipose chronic inflammation, glucose tolerance tests revealed that *ob/ob* mice with *EAT* gene transfer had better glucose tolerance and insulin sensitivity (Fig. 5H,I). Histological examination using H&E staining demonstrated that *EAT* gene transfer suppressed islet hypertrophy (Fig. 5J).

### *EAT* gene transfer blocks fatty liver development in *ob/ob* mice

**Figure 6:**
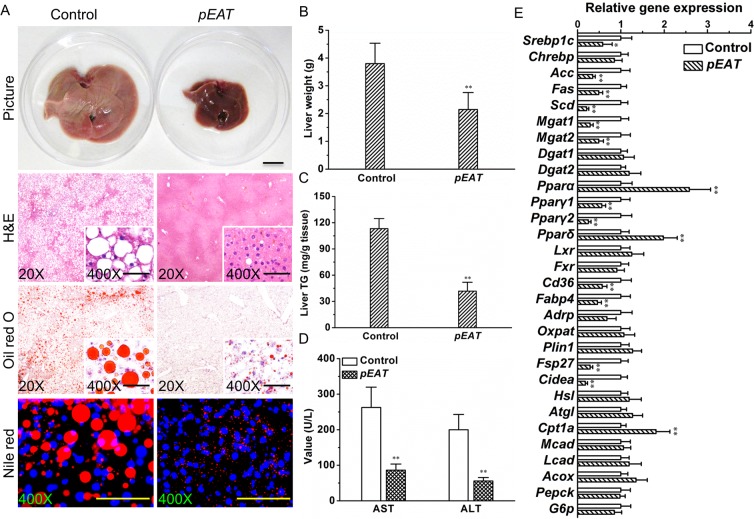
*EAT* gene transfer blocks fatty liver development in *ob/ob* mice. (**A**) Representative images of the liver and liver sections stained by H&E, Oil red O, or Nile red; Bar = 1 cm for gross image; Scale bar = 50 μm for tissue sections. (**B**) Liver weight. (**C**) Liver triglyceride. (**D**) Blood concentrations of AST and ALT. (**E**) mRNA levels of critical genes controlling lipid and glucose metabolism in the liver. Values in (**B–E**) represent average ± SD (n = 5). *P < 0.05 compared with the control; **P < 0.01 compared with the control.

To study the pathological changes in the liver of *ob/ob* mice with or without *EAT* gene transfer, we performed a series of histological examinations. Figure 6A shows that *EAT* gene transfer blocked the enlargement of liver and remarkably repressed hepatic fat deposition in the context of leptin deficiency. The average liver weight was ~43.5% lower in the treated mice compared to that of the control (Fig. 6B). *EAT* gene transfer repressed hepatic triglyceride content by ~63.1% (Fig. 6C). The mice injected with the *EAT* gene showed much lower concentrations of serum AST and ALT compared to the control (Fig. 6D), suggesting improved liver function. To explore the factors potentially involved, we determined transcription levels of a variety of key genes responsible for lipid and glucose metabolism in the liver. *EAT* gene transfer decreased expression levels of pivotal genes for hepatic lipid production and lipid droplet formation while elevating transcription levels of key factors responsible for fatty acid oxidation (Fig. 6E).

### Assessment of potential toxic effects of *EAT* gene transfer

**Figure 7:**
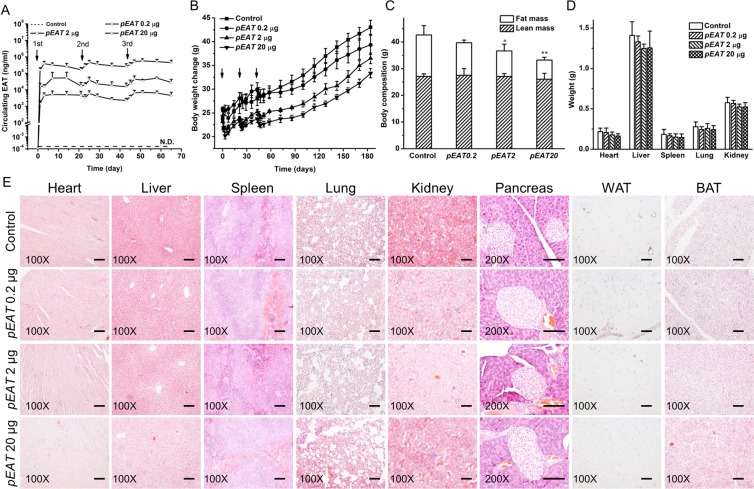
Long-term effects of repeated injection of *EAT* gene. (**A**) Blood levels of EAT protein post *EAT* gene transfer. (**B**) Weight gain of CD-1 mice on a standard chow. (**C**) Body composition of mice 180 days after initial hydrodynamic injection of pEAT plasmid. (**D**) Weights of internal organs. (**E**) Representative images of H&E staining of pivotal organ sections including the heart, liver, spleen, lungs, kidneys, pancreas, WAT and BAT; Scale bar = 50 μm. Values in (**A–D**) represent average ± SD (n = 5). *P < 0.05 compared with the control; **P < 0.01 compared with the control.

Repeated injection of different amounts of pEAT plasmid was performed on regular CD-1 mice fed a regular chow and its impacts on animals were examined. Three doses of pEAT plasmid at 0.2, 2.0, and 20.0 µg/mouse were employed. Three injections of the same dose were performed on days 0, 22, and 44, and blood samples were collected after each injection. Results in Fig. 7A show that blood concentration of EAT protein is dose dependent. The peak level of EAT protein was ~385 µg/ml in animals received 20.0 µg plasmid DNA, compared to ~52 µg/ml in animals injected with 2.0 µg, and ~5 µg/ml with 0.2 µg pEAT plasmid, respectively. EAT protein level declined slightly and regained the peak levels with the 2^nd^ and 3^rd^ injection. Animals grow slower with higher dose of plasmid injection, but growth is evident at later time (Fig. 7B). Body composition analysis shows the same amount of lean mass but different levels of fat mass depending on the amount of pEAT plasmid injected (Fig. 7C). At the end of the 6-month experiment, animals were euthanized and the internal organs were collected and weighted. Results summarized in Fig. 7D show similar weight of each of the internal organs including the heart, liver, spleen, lungs and the kidneys. No abnormal structures were identified by H&E staining of the tissue sections of the selected organs, and the WAT and BAT (Fig. 7E).

## Discussion

Obesity is a complex disease with severe comorbidities^21^. Targeting key components in obesity pathophysiology is expected to prevent and reverse obesity, and restore metabolic homeostasis. GLP-1 receptor has been considered as a target for treating this cluster of metabolic diseases due to its crucial role in regulating food intake and glucose metabolism^9^. A number of GLP-1 receptor agonists have been developed in recent years^22–24^. Most of these GLP-1 analogs were designed for enhanced stability and prolonged half-life^23^. Despite the improvements, the anti-inflammatory activity of conventional GLP-1 analogs is still limited^23,24^. In the current study, we placed Ex4, a potent agonist of GLP-1 receptor at the N-terminus of AAT, a protein with well-recognized anti-inflammatory and immune-modulatory activities^17–19^, and demonstrated that both AAT and Ex4 activities are preserved in our recombinant fusion protein, EAT. In line with the design, *EAT* gene transfer significantly reduced animal food intake and adipose inflammation in diet-induced and genetic obese mice. At the molecular level, *EAT* gene transfer decreased transcriptional levels of pivotal genes responsible for lipogenesis, lipid droplet formation and chronic inflammation in the liver. In addition, expression of the *EAT* gene also elevated expression of thermogenic genes in brown fat, indicating that this mechanism may also contribute to the weight loss observed in this study. In agreement with our findings, a recent study by Kooijman *et al*. provided elegant evidence demonstrating that Ex4 was able to activate BAT, thereby leading to a series of metabolic benefits in HFD-induced obese mice^25^.

Beneficial effects of *EAT* gene transfer on animals depend on a sufficient level of EAT protein in the blood. Hydrodynamic gene delivery was used in this study because of its high efficiency in gene delivery to the liver by a tail vein injection. The dose response curves show that sufficient level of EAT protein was achieved at 2.0 µg of pEAT plasmid per mouse, although significant level of EAT protein was also obtained with a lower dose at 0.2 µg/mouse. Persistent *EAT* gene expression in animals is largely due to the use of an albumin promoter in the pLIVE vector^26^. Increase in circulation half-life of EAT for Ex4 is another important factor for our design because Ex4 peptide is short lived in blood circulation^9^. Fusion of Ex4 to AAT creates a larger protein molecule that minimizes its renal elimination. Physiological benefits seen in the animals are likely due to persistent Ex4 activity in EAT that suppresses food intake and improves lipid metabolism and glucose homeostasis.

Chronic inflammation in the adipose tissue is an integral factor of obesity-associated metabolic dysregulations, particularly for insulin resistance^7^. Excess energy storage in fat causes adipocyte hypertrophy and apoptosis, leading to macrophage infiltration for removal of the damaged adipocytes, leading to development of crown-like structures, a sustained low-grade inflammation, a compromised metabolic homeostasis and insulin resistance^12,13,27^. Emerging evidence suggests that an imbalance between AAT and elastase activity contributes to this pathological development^28^. Both obese men and mice had reduced levels of serum AAT, and importantly, AAT transgenic mice were protected from HFD-induced inflammation and insulin resistance^28^. One previous study by Kalis *et al*. demonstrated that AAT treatment potentiated the effect of GLP-1 on insulin secretion and protected pancreatic β cells from cytokine-mediated apoptosis^29^. AAT has also been tested recently in clinic for the treatment of diabetes, owing to its anti-inflammatory and immune-modulatory activities^30–32^. In alignment with these previous studies, we demonstrated in the current study that EAT treatment suppressed adipocyte hypertrophy, blocked the formation of crown-like structures (Fig. 5E**)**, and thereby attenuated adipose inflammation and alleviated insulin resistance. It is important to note that insulin resistance is not always positively correlated with body weight. For example, without adipose inflammation, animals could become extremely obese without showing sign of insulin resistance^14–16^. Conversely, when affected by adipose metabolic dysfunction and inflammation, non-obese subjects in both humans and mice can exhibit insulin resistance^33,34^, suggesting that management of inflammation is a critical strategy in dealing with metabolic disorders.

The hepatic fat accumulation is controlled by a dynamic balance between liver lipid production and secretion, and communication with other organs^35^. In our study, *EAT* gene transfer reversed fatty liver in diet-induced obese mice and blocked hepatic steatosis in *ob/ob* mice. This effect can be explained, at least partially, by decreased hepatic lipogenesis, reduced lipid droplet formation, and improved liver-adipose tissue crosstalk. Though the underlying mechanism has not been fully understood, recent studies demonstrate the existence of GLP-1 receptors on hepatocytes, which directly implicates the involvement of GLP-1 in regulating lipid metabolism in the liver^36^. Extending these prior studies, we found in the current study that *EAT* gene transfer reduced expression of *Srebp1c* and its downstream target genes, including *Acc*, *Fas*, and *Scd* that are responsible for lipogenesis in the liver. The PPARγ pathway appears to play a central role in controlling hepatic lipid droplet development by regulating *CD36*, *FABP4*, *MGAT1* and lipid droplet surrounding proteins^37–39^. *EAT* gene transfer reduced transcription of all these crucial genes, indicating down-regulation of the PPARγ pathway as a potential mechanism of *EAT* gene therapy in blocking fatty liver development. In addition, the blocked hepatic steatosis may also be partially accounted for by improved liver-adipose tissue metabolic communication. It was reported earlier that the inter-organ crosstalk significantly affects lipid partitioning and storage in individual organs^40–42^. With the evidence presented in current study, it is conceivable that expression of the *EAT* gene can restore adipose dysfunction and promotes adipose storage capacity, thereby repressing lipid spillover and improving the inter-organ crosstalk, resulting in a blockade of fatty liver development.

Compared to conventional GLP-1 therapies developed recently which only target the GLP-1 receptors^22–24^, this newly-designed EAT therapy simultaneously targets multiple metabolic and inflammatory pathways. In addition to a possible application of *EAT* gene transfer to treatment and prevention of obesity and obesity-associated metabolic diseases, our data also show that overexpression of the *EAT* gene with three repeated injections at three different doses did not lead to aberrant histological changes in major organs and adipose tissues. Future studies could focus on a long-term evaluation to rule out any pathological effects of the *EAT* gene transfer. Studies with different animal species, especially using large animals, will confirm that the EAT effects seen in mice and demonstrated in the current study are not animal species dependent.

In conclusion, by focusing on the major components of obesity pathophysiology we created a novel fusion protein named *EAT* for pharmacological intervention of obesity and its associated metabolic disorders. We found in regular mice fed an HFD or standard chow, or diet-induced C57BL/6 obese and *ob/ob* mice that *EAT* gene transfer was able to generate various metabolic benefits, including prevention of HFD-induced weight gain, insulin resistance and fatty liver development, and therapeutic benefits of weight loss and improvement of metabolic homeostasis. The primary mechanisms underlying these beneficial effects are likely involved in a combined effect of Ex4 activity in suppressing food intake and improving metabolic homeostasis and the anti-inflammation effects of AAT in EAT protein. Our findings open a new avenue for a gene therapy-based treatment for obesity, diabetes, and NAFLD.

## Materials and Methods

### Plasmid construction and preparation

The Ex4 gene was designed according to its amino acid sequence and constructed using primers synthesized at Sigma-Aldrich (St. Louis, MO). The hAAT gene was cloned from the plasmid used in our previous study^43^. The EAT fusion gene was created by placing *Ex4* sequence at the 5′ end of hAAT gene with a linker sequence encoding GGGGS peptide. For *in vivo* transgene overexpression, we used a pLIVE plasmid vector purchased from Mirus Bio (Madison, WI). EAT gene was cloned into 3′ end of the albumin promoter at the multi-cloning sites of pLIVE vectors. The constructed plasmids with insert were verified by restriction enzyme digestion and DNA sequencing. Plasmid DNA was prepared by means of cesium chloride-ethidium bromide gradient centrifugation. The purity of the plasmid preparations was confirmed by OD_260/280_ ratio and by 1% agarose gel electrophoresis. The purified plasmids were dissolved in saline and stored at −80 °C until use.

### Preparation of EAT protein

We prepared EAT recombinant protein using transient overexpression in HEK293T cells purchased from ATCC (#CRL-3216). The cells were cultured using Dulbecco’s Modified Eagle’s medium (DMEM) containing 10% fetal bovine serum (FBS). The transfection was performed using polyethyleneimine (PEI, branched, Mw = 25,000 Da) as transfection reagent and conditions optimized for PEI:DNA ratio, and the amount of DNA per plate. The optimized conditions were used for EAT expression in T-175 flasks. For transfection, 40 μg of plasmid DNA were mixed with 120 μg of PEI and the complexes kept at room temperature for 10–20 min before being added to cells with ~60% confluency in a T-175 flask. The DMEM medium with 1% FBS was changed every 2 days for a total of 8 days and all culture medium was collected for EAT protein purification using two-step chromatography, including medium passing through a Nickel Affinity Column (#25215, Thermo-Scientific) and then an ion-exchange column (#17-0510-10, GE Healthcare Life Sciences) for further polishing. The purity of the final product was determined using 10% SDS-PAGE loaded with 2–8 μg recombinant protein per lane and stained with a kit (#1610803) purchased from Bio-Rad. The purified EAT protein was further verified using Western blotting with an antibody recognizing hAAT (#A0409, Sigma-Aldrich).

### Protease inhibition assay

The fluorescence-based elastase inhibitor assay kit (#K782) was purchased from BioVision (San Francisco, CA). The enzymatic assay was carried out following the instruction from the kit. In brief, purified EAT proteins was diluted at different concentrations in the range of 0.0128–1000 μg/ml. The total reaction volume was 100 μl, and the excitation and emission wavelength were 400 nm and 505 nm, respectively. The mixtures were incubated at 37 °C for 30 min before fluorescence intensity measurement using a Fluoro-Max 4 fluorimeter (Horiba Jobin Yvon).

### Animals and treatments

All procedures performed on animals were approved by the Institutional Animal Care and Use Committee at the University of Georgia, Athens, Georgia (protocol number, #A2014-07-008-Y1-A0). All experiments were performed in accordance with relevant guidelines and regulations. The high-fat diet (60% energy from fat) used in this study was purchased from Bio-Serv (#F3282, Frenchtown, NJ). Male C57BL/6 mice and CD-1 mice were purchased from Charles River Laboratories (Wilmington, MA) and fed a high-fat diet for 12–16 weeks to induce obesity. Leptin deficient *ob/ob* mice were purchased from the Jackson Laboratory (Bar Harbor, ME) and kept on standard chow. Animals were housed under standard conditions with a 12 h light-dark cycle. Hydrodynamic gene transfer in obese mice was performed according to an established procedure with some modification^44,45^. Briefly, 2.0–2.5 ml saline solution (equivalent to 8% lean mass of obese mice) containing different amounts of plasmid DNA was injected into the tail vein of mouse within 5–8 s. Food consumption was determined by measuring the difference between the amount provided and the amount left twice weekly. Daily food intake per mouse was calculated based on the amount consumed divided by time and the number of mice per cage. Body composition was analyzed using an EchoMRI-100 (Echo Medical Systems, Houston, TX).

### Histological examinations and liver triglyceride determination

Liver samples were collected and fixed in 10% neutral buffered formalin (NBF). After dehydration using gradient ethanol, the samples were processed twice using xylene and embedded in paraffin. Tissue sections (6 μm in thickness) were made, spread on a slide and baked at 37 °C for 2 h. The slides were stained with H&E, mounted with Permount medium (Fisher Scientific), and examined under an optical microscope (ECLIPSE Ti, Nikon). Image quantification was carried out using NIS-Elements imaging platform from Nikon Instruments Inc. (Melville, NY). For Oil red O and Nile red staining, frozen liver samples were cut at 8 μm in thicknesses and fixed using 10% NBF. Liver triglyceride content was determined following a previously reported method with some modification^46^. In brief, freshly collected liver tissues (100–200 mg) were homogenized in a mixture of chloroform and methanol (2:1) and tissue homogenates incubated overnight at 4 °C. The mixture was centrifuged at 12,000 rpm for 20 min and supernatant collected. The collected fractions were dried and lipids re-dissolved in 1% Triton X-100. The triglyceride concentration was determined using a commercial kit (#TR22203) from Thermo-Scientific (Waltham, MA).

### Evaluation of glucose tolerance and insulin sensitivity

For the glucose tolerance test (GTT), mice were injected intraperitoneally with glucose at 2 g/kg body weight after 6 h fasting. Blood samples (10 μl each) were taken from the tail vein at varying time points and glucose concentrations were determined using glucose test strips and a TRUEtrack glucose meter purchased from Nipro Diagnostics Inc (Fort Lauderdale, FL). For the insulin tolerance test (ITT), mice fasted for 4 h and blood glucose levels were measured after an intraperitoneal injection of insulin (0.75 U/kg) purchased from Eli Lilly (Indianapolis, IN) using blood samples collected from the tail vein at different time points.

### Determination of blood concentrations of insulin, TNFα, IL6, AST, and ALT

Plasma samples were prepared by centrifugation of freshly collected blood in a heparin coated tube at 4,000 rpm for 5 min, and kept frozen at −80 °C until use. Insulin concentrations were determined using an insulin ELISA kit (#10-1247-01) purchased from Mercodia Developing Diagnostics (Winston-Salem, NC). Blood levels of TNFα and IL6 were determined using ELISA kits from eBioscience (San Diego, CA). AST and ALT levels were determined using biochemical kits purchased from Thermo-Scientific (Waltham, MA). All of the measurements were performed following the protocol provided by the manufacturer.

### Gene expression analysis

Total RNA was isolated using the TRIZOL reagent (Life Technologies, Grand Island, NY) for liver samples or RNeasy Lipid Tissue Mini Kit (QIAGEN, Valencia, CA) for adipose tissue samples according to the manufacturers’ protocols. One microgram of total RNA was used for the first strand cDNA synthesis using a First-strand cDNA Synthesis kit purchased from OriGene (Rockville, MD). Real time PCR was performed on an ABI StepOne Plus Real Time PCR system (Foster City, CA) using PerfeCTa® SYBR® Green FastMix (Quanta BioSciences, Gaithersburg, MD) as the indicator. Primers were synthesized at Sigma (St. Louis, MO). Melting curve analysis of all real-time PCR products was conducted and showed a single DNA duplex. The primer sequences employed are summarized in Supplementary Table 1. The data were analyzed using the ΔΔCt method^47^.

### Determination of blood concentration of EAT protein

Standard ELISA protocol was followed to determine EAT protein concentration^43^. In brief, a rabbit anti human AAT antibody was used to coat the ELISA plate overnight and blocked for 1 h with blocking buffer (4% BSA in PBS-Tween buffer). Serum prepared from animals was diluted serially with 1% BSA in PBS-Tween 80 buffer and added to each well of the ELISA plate and incubated for 1 h. After washing, biotinylated goat anti-human AAT polyclonal antibody (1:1000 dilution in 1% BSA Tween 80 buffer) was added and incubated for 1 h at room temperature. After washing, streptavidin-horseradish peroxidase conjugate (1:50,000 dilution) was added and incubated for 1 h. The substrate solution (3,3′, 5,5′-tetramethylbenzidine) was added and the plate was read at 450 nm in an ELISA reader. The AAT concentration was calculated based on a standard curve established in each plate using known amount of pure hAAT. With the exception of blocking buffer (200 µl/well) and washing buffer (200 µl/well), the sample volume used was 100 µl/well.

### Statistics

Statistical analysis was performed by using the Student’s *t* test and one-way ANOVA. A *P* value below 0.05 (*P* < 0.05) was considered significantly different. All results were expressed as the mean ± SD.

## Supplementary information

Supplemantery Information
