# Supplementary material for: Controlling Obesity and Metabolic Diseases by Hydrodynamic Delivery of a Fusion Gene of Exendin-4 and α1 Antitrypsin
Source: Sci Rep. 2019 Sep 17;9:13427. doi: 10.1038/s41598-019-49757-y (PMC6748963; doi:10.1038/s41598-019-49757-y)

Controlling Obesity and Metabolic Diseases by Hydrodynamic Delivery of a Fusion Gene of Exendin-4 and α1-Antitrypsin

Mingming Gao and Dexi Liu

Supplementary Table 1. Primer sequences employed in q-PCR.

Gene name	Forward primer sequence (F)	Reverse primer sequence (R)
Acc	F: ATGGGCGGAATGGTCTCTTTC	R: TGGGGACCTTGTCTTCATCAT
Acox	F: CCGCCACCTTCAATCCAGAG	R: CAA GT TCTCGATTTCTCGACGG
Adrp	F: GACCTTGTGTCCTCCGCTTAT	R: CAACCGCAATTTGTGGCTC
Atgl	F: GGATGGCGGCATTTCAGACA	R: CAAAGGGTTGGGTTGGTTCAG
Cd11b	F: ATGGACGCTGATGGCAATACC	R: TCCCCATTCACGTCTCCCA
Cd11c	F: CTGGATAGCCTTTCTTCTGCTG	R: GCACACTGTGTCCGAACTCA
Cd36	F: ATGGGCTGTGATCGGAACTG	R: GTCTTCCCAATAAGCATGTCTCC
Chrebp	F: AGATGGAGAACCGACGTATCA	R: ACTGAGCGTGCTGACAAGTC
Cidea	F: TGACATTCATGGGATTGCAGAC	R: GGCCAGTTGTGATGACTAAGAC
Cpt1a	F: CTCCGCCTGAGCCATGAAG	R: CACCAGTGATGATGCCATTCT
Dgat1	F: TCCGTCCAGGGTGGTAGTG	R: TGAACAAAGAATCTTGCAGACG
Dgat2	F: GCGCTACTTCCGAGACTACTT	R: GGGCCTTATGCCAGGAAACT
Dio2	F: AATTATGCCTCGGAGAAGACCG	R: GGCAGTTGCCTAGTGAAAGGT
Elovl3	F: TTCTCACGCGGGTTAAAAATGG	R: GAGCAACAGATAGACGACCAC
F4/80	F: TGACTCACCTTGTGGTCCTAA	R: CTTCCCAGAATCCAGTCTTTCC
Fabp4	F: AAGGTGAAGAGCATCATAACCC	R: TCACGCCTTTCATAACACATTCC
Fas	F: GGAGGTGGTG ATAGCCGGTAT	R: TGGGTAATCCATAGAGCCCAG
Fsp27	F: ATGGACTACGCCATGAAGTCT	R: CGGTGCTAACACGACAGGG
Fxr	F: GCTTGATGTGCTACAAAAGCTG	R: CGTGGTGATGGTTGAATGTCC
G6p	F: CGACTCGCTATCTCCAAGTGA	R: GTTGAACCAGTCTCCGACCA
Gapdh	F: AGGTCGGTGTGAACGGATTTG	R: TGTAGACCATGTAGTTGAGGTCA
Hsl	F: CCAGCCTGAGGGCTTACTG	R: CTCCATTGACTGTGACATCTCG
Il6	F: TAGTCCTTCCTACCCCAATTTCC	R: TTGGTCCTTAGCCACTCCTTC
Lcad	F: TCTTTTCCTCGGAGCATGACA	R: GACCTCTCTACTCACTTCTCCAG
Lpl	F: GGGAGTTTGGCTCCAGAGTTT	R: TGTGTCTTCAGGGGTCCTTAG
Lxr	F: CTCAATGCCTGATGTTTCTCCT	R: TCCAACCCTATCCCTAAAGCAA
Mcad	F: GGGTTTAGTTTTGAGTTGACGG	R: CCCCGCTTTTGTCATATTCCG
Mcp1	F: TTAAAAACCTGGATCGGAACCA	R: GCATTAGCTTCAGATTTACGGGT
Mgat1	F: TGGTGCCAGTTTGGTTCCAG	R: TGCTCTGAGGTCGGGTTCA
Mgat2	F: TGGGAGCGCAGGTTACAGA	R: CAGGTGGCATACAGGACAGA
Oxpat	F: TGTCCAGTGCTTACAACTCGG	R: CAGGGCACAGGTAGTCACAC
Pepck	F: CTGCATAACGGTCTGGACTTC	R: CAGCAACTGCCCGTACTCC
Pgc1α	F: TATGGAGTGACATAGAGTGTGC	R: CCACTTCA ATCCACCCAGAAAG
Plin1	F: GGGACCTGTGAGTGCTTCC	R: GTATTGAAGAGCCGGGATCTTTT
Pparα	F: AGAGCCCCATCTGTCCTCTC	R: ACTGGTAGTCTGCAAAACCAAA
Pparγ1	F: GGAAGACCACTCGCATTCCTT	R: GTAATCAGCAACCATTGGGTCA
Pparγ2	F: TCGCTGATGCACTGCCTATG	R: GAGAGGTCCACAGAGCTGATT
Pparδ	F: TCCATCGTCAACAAAGACGGG	R: ACTTGGGCTCAATGATGTCAC
Scd	F: TTCTTGCGATACACTCTGGTGC	R: CGGGATTGAATGTTCTTGTCGT
Srebp1c	F: GCAGCCACCATCTAGCCTG	R: CAGCAGTGAGTCTGCCTTGAT
Tnfa	F: CCCTCACACTCAGATCATCTTC	R: GCTACGACGTGGGCTACAG
Ucp1	F: AGGCTTCCAGTACCATTAGGT	R: CTGAGTGAGGCAAAGCTGATTT

Uncropped image for Figure 1B

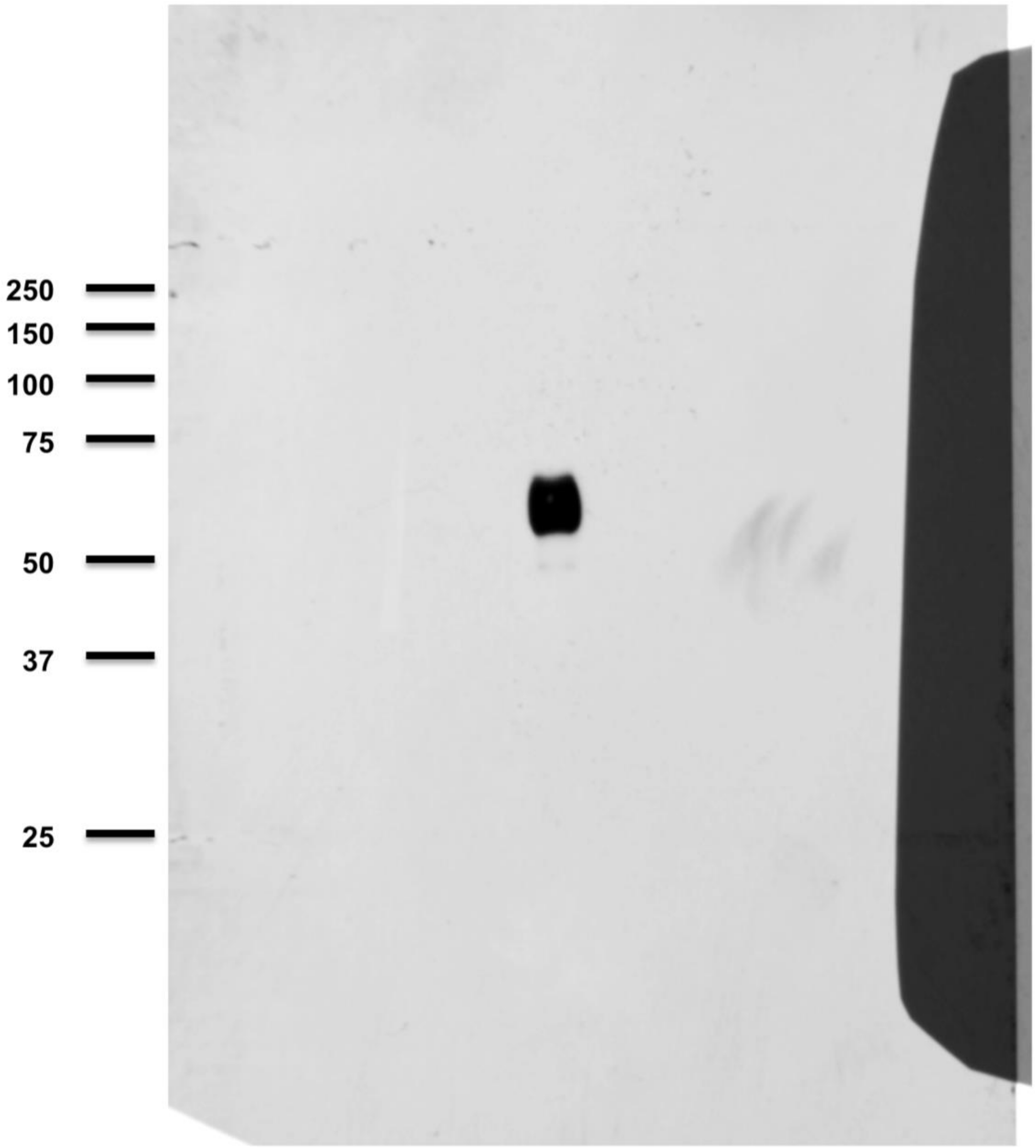

Supplement: Supplementary file 1 — Supplemantery Information [file 41598_2019_49757_MOESM1_ESM.pdf]
